# Strain and bioprocess improvement of a thermophilic anaerobe for the production of ethanol from wood

**DOI:** 10.1186/s13068-016-0536-8

**Published:** 2016-06-16

**Authors:** Christopher D. Herring, William R. Kenealy, A. Joe Shaw, Sean F. Covalla, Daniel G. Olson, Jiayi Zhang, W. Ryan Sillers, Vasiliki Tsakraklides, John S. Bardsley, Stephen R. Rogers, Philip G. Thorne, Jessica P. Johnson, Abigail Foster, Indraneel D. Shikhare, Dawn M. Klingeman, Steven D. Brown, Brian H. Davison, Lee R. Lynd, David A. Hogsett

**Affiliations:** Mascoma Corporation, 67 Etna Rd, Lebanon, NH 03766 USA; Thayer School of Engineering, Dartmouth College, 14 Engineering Drive, Hanover, NH 03755 USA; Verdezyne, Carlsbad, CA USA; Novogy Inc, Cambridge, MA 02138 USA; Bioenergy Science Center, Oak Ridge, TN USA; Genzyme, Cambridge, MA USA; Myriant Corporation, Quincy, MA USA; Washington, DC, USA; Nalco Champion, Houston, TX USA; Biosciences Division, Oak Ridge National Laboratory, Oak Ridge, TN USA; Novozymes Inc, Davis, CA USA

**Keywords:** Cellulosic ethanol, Consolidated bioprocessing, Organism development, Metabolic engineering, Bioprocess development, Thermophilic bacteria

## Abstract

**Background:**

The thermophilic, anaerobic bacterium *Thermoanaerobacterium saccharolyticum* digests hemicellulose and utilizes the major sugars present in biomass. It was previously engineered to produce ethanol at yields equivalent to yeast. While saccharolytic anaerobes have been long studied as potential biomass-fermenting organisms, development efforts for commercial ethanol production have not been reported.

**Results:**

Here, we describe the highest ethanol titers achieved from *T. saccharolyticum* during a 4-year project to develop it for industrial production of ethanol from pre-treated hardwood at 51–55 °C. We describe organism and bioprocess development efforts undertaken to improve ethanol production. The final strain M2886 was generated by removing genes for exopolysaccharide synthesis, the regulator *perR*, and re-introduction of phosphotransacetylase and acetate kinase into the methyglyoxal synthase gene. It was also subject to multiple rounds of adaptation and selection, resulting in mutations later identified by resequencing. The highest ethanol titer achieved was 70 g/L in batch culture with a mixture of cellobiose and maltodextrin. In a “mock hydrolysate” Simultaneous Saccharification and Fermentation (SSF) with Sigmacell-20, glucose, xylose, and acetic acid, an ethanol titer of 61 g/L was achieved, at 92 % of theoretical yield. Fungal cellulases were rapidly inactivated under these conditions and had to be supplemented with cellulosomes from *C. thermocellum*. Ethanol titers of 31 g/L were reached in a 100 L SSF of pre-treated hardwood and 26 g/L in a fermentation of a hardwood hemicellulose extract.

**Conclusions:**

This study demonstrates that thermophilic anaerobes are capable of producing ethanol at high yield and at titers greater than 60 g/L from purified substrates, but additional work is needed to produce the same ethanol titers from pre-treated hardwood.

## Background

Biotechnology for the conversion of biomass to fuels has the potential to reduce the need for carbon-intensive fossil fuels, but must be cost-competitive to be commercialized. Ethanol is the first commercial cellulosic biofuel and the logical proving ground for innovations aimed at reducing production costs. To be cost-competitive, an improved process must generate ethanol at high yield. Sufficiently high ethanol titers, generally at or above 40 g/L [1, 2], are also required to avoid high costs for fermentation and distillation. The upper limit of ethanol titer that can be achieved with lignocellulosic feedstocks is considerably lower than can be achieved from starch due to the lower fraction of fermentable sugar and materials handling issues [2]. As a result, both near-term and futuristic designs for cellulosic ethanol plants often involve ethanol titers in the range of 50–60 g/L [3, 4].

Thermophilic, anaerobic bacteria exhibit distinctively high rates of cellulose and plant cell wall solubilization [2, 5], with fermentation of cellulose and hemicellulose usually carried out by different species. *Thermoanaerobacterium saccharolyticum* ferments xylan, the main polymer in hemicellulose, and also utilizes all other major biomass sugars, including cellobiose, glucose, mannose, xylose, galactose, and arabinose. This microorganism does not, however, ferment cellulose to any significant degree. Organic fermentation products from wild-type strains of *T. saccharolyticum* strains include ethanol, acetic acid, and lactic acid. By deleting the genes encoding lactate dehydrogenase, phosphotransacetylase, and acetate kinase, an engineered strain was developed that produces ethanol at greater than 90 % of theoretical yield, equivalent to yeast and other homoethanologens [6]. *T. * saccharolyticum is naturally competent and recombinogenic, making genetic manipulation relatively easy [7]. The genome sequence and other genomic resources have been recently published [8]. Beginning with a homoethanologenic strain of *T. saccharolyticum*, Shaw et al. [9] achieved an ethanol titer of 54 g/L by introducing genes encoding urease and using urea as the nitrogen source. To our knowledge, this is the highest titer of produced ethanol reported for a thermophilic bacterium.

The US Department of Energy Biomass Program and Mascoma Corporation funded a 4-year project to develop *T. saccharolyticum* as a biocatalyst for the production of ethanol from pre-treated hardwood [10]. The two main components of the project were organism and bioprocess development activities. Organism development efforts were aimed at generating strains to produce high ethanol titers in the presence of inhibitors found in pre-treated biomass, using a combination of rational genetic engineering, classical mutagenesis/selection, and genome-scale resources. Bioprocess development efforts were aimed at meeting specific performance targets using optimization of media, enzyme addition, growth on hardwood substrates, and process integration. The two activities were pursued in parallel and subsequently brought together to achieve high ethanol titers, first with purchased model substrates, nutrients and inhibitors, and then progressing to pre-treated hardwood.

The original vision was to use *T. saccharolyticum* in a simultaneous saccharification and fermentation (SSF) process configuration. Since the fermentation temperature of *T. saccharolyticum* matches the optimal temperature for many fungal cellulases, we expected to add less cellulase than would otherwise be necessary. However, we discovered mid way through the project that commercial fungal cellulases are reversibly inactivated by the low-redox fermentation conditions [11]. A related project aimed to express cellulases in *T. saccharolyticum* [12], but the maximal expression and secretion levels were insufficient. Ultimately, cellulosome preparations from *C. thermocellum* were used to overcome the limitations of fungal cellulase, as described below. We also describe the rationale for directed strain modifications and the sequence-level effects of selections and adaptations. Finally, we present performance data for both model substrates and conditions more representative of an industrial process.

## Results and discussion

### Strain development

We previously described a method to perform markerless genetic manipulations in *T. saccharolyticum*. It is “markerless” in so far as it allows the removal of the antibiotic resistance genes (i.e., markers) after they are used [13]. The method is based on negative selection against the presence of the *pta* and *ack* genes with chloroacetate. It was used to eliminate lactate and acetate production in wild-type strain JW/SL-YS485 (DSM 8691), creating homoethanologen strain M355 [13]. This strain was then subjected to multiple rounds of nitrosoguanidine mutagenesis and screening for high ethanol titers in the presence of an enzymatic hydrolysate from pre-treated hardwood by Panlabs Biologics in Taiwan.

The 14 top-performing strains from that effort (M796–M809) were mixed and used as inoculum into a cytostat containing a mixture of inhibitory chemicals found in pre-treated hardwood and 20 g/L ethanol. A cytostat is a cell density-regulated continuous culture that uses a highly sensitive flow cytometer to measure cell density, allowing the culture to be maintained continuously at low cell density and fast growth rates [14]. A single clone was isolated from the cytostat and designated M863 (Table 1).

**Table 1:** Strains used in present study

Strain #	Description of genetic manipulation	Genotype
M355	Markerless KO of genes for acetate and lactate production	*pta*/*ack*(−) L-*ldh*(−)
M795–M809	Mix of strains generated by Panlabs using NTG mutagenesis and selection on wood hydrolysate	*pta*/*ack*(−) L-*ldh*(−)
M863	Selection in Cytostat with synthetic mix of inhibitors and 20 g/L ethanol	*pta*/*ack*(−) L-*ldh*(−)
M1151	Addition of urease, fix of *metE* and markerless KO of Tsac_0795	*pta*/*ack*(−) L-*ldh*(−) Tsac_0795(−) urease(+) *metE*(+)
M1291	Markerless KO of putative EPS operon (phosphoglucomutase, UDP-G1P transferase, transmembrane protein, near gene Tsac_1471)	*pta*/*ack*(−) L-*ldh*(−) Tsac_0795(−) urease(+) *metE*(+) EPSoperon(−)
M1442^a^	Selection in auxostat for fast growth in glucose, xylose, arabinose, and acetic acid	*pta*/*ack*(−) L-*ldh*(−) Tsac_0795(−) urease(+) *metE*(+) EPSoperon(−)
M2476	Markerless KO of *perR*	*pta*/*ack*(−) L-*ldh*(−) Tsac_0795(−) urease(+) *metE*(+) EPSoperon(−) *perR*(−)
M2886	Insertion of *pta*/*ack*-KanR into methylglyoxal synthase *mgs*	*pta*/ack(−) L-*ldh*(−) Tsac_0795(−) urease(+) *metE*(+) EPSoperon(−) *perR*(−) *mgs::pta*/*ack*-KanR

^a^Strain M1442 is also known as LL1049

Using an approach as described previously [15], a library of clones was created that positioned random pieces of *T. saccharolyticum* DNA down-stream from a strong promoter integrated into the *T. saccharolyticum* chromosome, with the expectation that overexpression of some genes would lead to improved inhibitor tolerance. The library was selected on solid or liquid media containing extracts from pre-treated hardwood. Sequencing the inserts showed that 19 out of 23 selected clones had the *pta/ack* gene pair inserted. This was surprising, since the strain had been engineered to eliminate acetate production by the removal of these genes. Also intriguing, the library-selected strains did not produce wild-type levels of acetate and the *pta/ack* genes confer inhibitor tolerance even without net acetate production. An investigation of this result is published elsewhere [16].

A related cloning strategy was used to create a random deletion library in *T. saccharolyticum* which was subjected to selection in the cytostat with mixed inhibitors and in auxostat cultures with extracts of pre-treated hardwood. An auxostat is a continuous culture in which the feed rate is indirectly coupled to growth rate. In this case, growth caused a drop in pH from the uptake of ammonia, which was countered by automatic addition of a base solution to maintain a constant pH mixed with growth-inhibitory extract. The dilution rates of both cytostats and auxostats are proportional to growth, but in practice, the auxostat has a higher cell density and slower growth rate. The deletion library yielded a wider assortment of genotypes than the overexpression library, but both cytostat and auxostat selected for clones with a deletion in the gene Tsac_0795, encoding a possible helicase or protein kinase. Further strain improvement consisted of a knockout of Tsac_0795, while simultaneously adding beneficial genes. The urease genes from *C. thermocellum* were inserted in place of Tsac_0795 to allow the use of urea as nitrogen source, which was shown to result in higher ethanol titers [9]. Also inserted at the same locus was the *metE* gene from *Caldicellulosiruptor kristjanssonii* to restore vitamin B-12-independent methionine synthesis, compensating for the disrupted native *metE* gene in *T. saccharolyticum*.

We next deleted a 4-gene putative operon that appeared to be related to exopolysaccharide synthesis: genes Tsac_1474-Tsac_1477, annotated as phosphoglucomutase, NGN domain-containing protein, UTP-glucose-1-phosphate uridylyltransferase, and lipopolysaccharide biosynthesis protein. The resulting strain M1291 produced more ethanol than its parent strain M1151 (Table 2), possibly due to diversion of intracellular glucose from anabolism (polymerization) to catabolism (glycolysis). This strain was then selected for rapid growth on mixed sugars by growing it for 425 h in a pH-controlled auxostat containing xylose, glucose, arabinose, and acetic acid, at growth rates from 0.09 to 0.37 h^−1^.

**Table 2:** Production of ethanol from 60 g/L cellobiose, 90 g/L maltodextrin by strains M1151, M1291, and M1442 in bottles

Medium	Strain	Final ethanol (g/L)	SD (g/L)
TSC-3	M1151	61.0	1.9
TSC-3	M1291	65.1	2.0
TSC-3	M1442	70.1	1.0
TSC-4	M1442	60.0	0.4

The next modification consisted of a markerless deletion of the regulatory gene *perR* to generate strain M2476. PerR is a repressor of oxidative stress response genes, and its deletion has been shown to increase aerotolerance in *C. acetobutylicum* [17]. Microarray studies with *T. saccharolyticum* looking at the response to inhibitors in pre-treated hardwood suggested an oxidative challenge [8], and we reasoned that overexpression of the *perR* regulon would increase tolerance to these inhibitors. Indeed, knockout mutants of *perR* in *T. saccharolyticum* (gene Tsac_2491) produced more ethanol than their parent from inhibitory concentrations of pre-treated hardwood hemicellulose extract (data not shown). The bacterium was also able to survive up to 4 h of air exposure on a pertri plate without an observable drop in viability. In contrast, the parent began to lose viability after 1 h under the same conditions.

Finally, the gene-encoding methylglyoxal synthase (*mgs,* Tsac_2114) was deleted by insertion of the kanamycin resistance marker and the *pta/ack* genes, creating strain M2886. While *T. saccharolyticum* grows well in high levels of starch and cellobiose, it is inhibited by monosaccharides at concentrations greater than 40 g/L. Glucose toxicity has been shown to correlate with the production of methylglyoxal [18]. The strain M2886 grew at 100 g/L glucose and produced more ethanol from pre-treated hardwood hydrolysate than other candidate strains.

It should be noted that many other approaches, both rational and selection-based, were tested in addition to those that were used to generate strain M2886. Strain benchmark tests were performed throughout its development with up to 30 strains at a time in standardized conditions to identify the best-performing strains and eliminate less-beneficial approaches. The benchmark tests comprised bottle cultures with high sugars (e.g., Table 2), SSFs on purified cellulose or challenges with inhibitory levels of pre-treated hardwood extracts, with maximum ethanol titer being the key metric. The strain lineage described here represents the top-performing modifications from each round of strain evaluation.

### Resequencing results

Strains M863, M1442, and M2886 were resequenced by Illumina sequencing, and compared to the wild-type JW/SL-YS485 genome sequence. Strain LL1025, which is another clone of JW/SL-YS485, was also sequenced as a control. Small-scale sequence variations are shown in Table 3. Seven sequence differences were found in all four strains, including LL1025 (rows 1–7), indicating possible errors in the Genbank genome sequence. Rows 8–10 show differences detected only in strain M863. Since the later strains were descended from M863, they should also contain these differences yet do not, suggesting that they are artifacts. A total of 16 small variations were detected in strain M863 and the later strains, likely arising during the extensive selections that took place to generate M863. These include mutations in the genes for the bifunctional acetaldehyde/alcohol dehydrogenase gene *adhE*, and in the *hfs* hydrogenase cluster, whose effect on ethanol production has been described elsewhere [16]. Selection in continuous culture preceding the isolation of M1442 resulted in nine mutations compared to the parent strain. Five additional small mutations arose in generating strain M2886.

**Table 3:** Genomic resequencing results from strains in the present study: small-scale sequence variations and their occurrence in each strain

Row	Gene	Nucleotide	Type	Reference	Change	Non-synonymous	Amino acid change	Percent of sequencing reads				Description
								YS485 (WT)	M863	M1442	M2886	
1	Tsac_0902	944418	Deletion	G	–	Intergenic		99	95	98	99	Hypothetical protein
2	Tsac_1176	1217778	Insertion	–	A	Intergenic		100	100	99	100	Histidinol dehydrogenase, prokaryotic-type
3	Tsac_1332	1388136	Insertion	–	A	Intergenic		100	100	99	100	Hypothetical protein
4	Tsac_1641	1717467	Insertion	–	T	Yes	Tyr 18 FS	100	100	100	100	MerR family transcriptional regulator
5	Tsac_2098	2143046	Insertion	–	A	Intergenic		100	100	100	100	Peptidase S11, d-alanyl-d-alanine carboxypeptidase A
6	Tsac_2308	2368691	Deletion	A	–	Intergenic		100	100	100	100	HEPN domain protein
7	Tsac_2828	21 (megaplasmid)	MNV	GA	AG	Yes	Lys 104 Glu	96	88	97	95	Hypothetical protein, on megaplasmid CP003185
8	Tsac_0096	92464	Insertion	–	T	Intergenic		100	PWT	99	100	LuxR family transcriptional regulator
9	Tsac_0303	322547	Insertion	–	A	Intergenic		99	40	99	92	Fibronectin type III domain protein
10	Tsac_0902	944612	SNV	A	T	No		PWT	49	PWT	PWT	Putative transposase YhgA family protein
11	Tsac_0032	30909	Insertion	–	A	Yes	Gly 177 FS	PWT	100	99	100	PTS system,* N*-acetylglucosamine-specific IIBC subunit
12	Tsac_0079	79429	Deletion	A	–	Yes	Asn 86 FS	PWT	74	99	100	Uncharacterised conserved protein UCP018688
13	Tsac_0144	150053	SNV	C	T	Intergenic		PWT	98	99	100	4-Deoxy-l-threo-5-hexosulose-uronate ketol-isomerase
14	Tsac_0163	177035	SNV	A	C	Yes	Phe 104 Val	PWT	100	100	100	Altronate oxidoreductase
15	*adhE* Tsac_0416	448858	SNV	G	A	Yes	Gly 544 Asp	PWT	98	100	100	Bifunctional alcohol/aldehyde dehydrogenase
16	Tsac_0838	884294	Deletion	T	–	Yes	Ser 102 FS	PWT	85	99	100	Protein of unknown function DUF324
17	Tsac_0948	993551	SNV	A	T	Yes	Phe 361 Tyr	PWT	100	100	100	Spore germination protein
18	*hfsB* Tsac_1551	1627813	Deletion	A	–	Yes	Lys 146 FS	PWT	100	99	100	Hydrogenase large subunit domain protein
19	*hfsD* Tsac_1553	1630591	SNV	A	T	Yes	Arg 107 Ser	PWT	98	100	100	Iron hydrogenase, large subunit, C-terminal
20	Tsac_1726	1800969	Insertion	–	A	Yes	Tyr 454 FS	PWT	100	100	100	Serine/threonine protein kinase with PASTA sensor(s)
21	*pta* Tsac_1744^a,b^	1814403	Insertion	–	G	Yes	Ile 3 FS	PWT	100	97	PWT	Phosphate acetyltransferase
22	Tsac_1782	1848944	Deletion	G	–	Yes	Glu 180 FS	PWT	100	99	100	Flagellar motor switch protein FliG
23	Tsac_2119	2160615	SNV	C	T	Yes	Gly 488 Glu	PWT	97	100	100	Penicillin-binding protein 2
24	Tsac_2196	2239931	SNV	G	A	No		PWT	100	99	100	Phenylalanyl-tRNA synthetase, class IIc, alpha subunit
25	Tsac_2229	2273080	SNV	A	G	Yes	Ser 283 Pro	PWT	82	99	100	Bacterial inner-membrane translocator
26	Tsac_2476	2526425	SNV	A	G	No		PWT	94	100	100	RNA polymerase, sigma 70 subunit, RpoD subfamily
27	Tsac_0535	559915	SNV	C	T	Yes	Gly 257 Asp	PWT	PWT	100	100	Cyanophycin synthetase
28	Tsac_0795^a^	835013	Deletion	A	–	Yes	Ile 1000 FS	PWT	PWT	100	100	SNF2-related protein
29	Tsac_1054	1089642	SNV	T	C	Intergenic		PWT	PWT	100	100	Thioredoxin reductase
30	Tsac_1296^a^	1356848	SNV	A	G	Yes	Glu 105 Gly	PWT	PWT	100	100	UDP-glucose 4-epimerase
31	Tsac_1419	1491395	SNV	C	A	Yes	Ala 187 Asp	PWT	PWT	99	100	ATPase, F0 complex, subunit A
32	Tsac_1477	1557984	Insertion	–	G	Yes	Ile 267 FS	PWT	PWT	100	100	Lipopolysaccharide biosynthesis
33	Tsac_1712	1787698	SNV	C	A	Yes	Pro 161 Thr	PWT	PWT	100	100	Diaminopimelate epimerase
34	Tsac_2390	2460517	SNV	C	T	Yes	Leu 311 Phe	PWT	PWT	99	100	Hypothetical protein
35	Tsac_2507	2555785	SNV	G	A	Yes	Gln 840 stop	PWT	PWT	100	100	PTS system transcriptional activator
36	Tsac_1486	1566717	MNV	AA	TT	Intergenic		PWT	PWT	PWT	100	Flavodoxin/nitric oxide synthase
37	Tsac_1725	1799018	SNV	G	A	Yes	Ala 50 Thr	PWT	PWT	PWT	100	Protein serine/threonine phosphatase
38	Tsac_2189	2229111	Insertion	–	T	Yes	Asp 144 FS	PWT	PWT	PWT	100	Two component transcriptional regulator, LuxR family
39	*perR* Tsac_2491^a^	2541836	Deletion	C	–	Yes	Ser 113 FS	PWT	PWT	PWT	100	Ferric uptake regulator, Fur family
40	*perR* Tsac_2491^a^	2541840	Deletion	T	–	Yes	Tyr 115 FS	PWT	PWT	PWT	100	Ferric uptake regulator, Fur family
41	Tsac_0622	659800	SNV	T	G	Intergenic		PWT	PWT	PWT	45	Hypothetical protein
42	Tsac_0634	677092	SNV	T	G	Intergenic		PWT	PWT	PWT	41	Glucokinase, ROK family

Where the sequence analysis software detected the mutation in greater than 20 % of reads, the percent of reads with the mutation is given. Otherwise, the percent of sequencing reads is not calculated

*SNV* single nucleotide variation, *FS* frame shift, *PWT* presumptively wild type, *MNV* multiple nucleotide variation

^a^These variations occurred in the small residual sequences that were not removed when the genes were knocked out

^b^The pta/ack genes were re-introduced into strain M2886

Table 4 shows nine larger-scale variations that were identified in the resequencing data. Six of these were the engineered deletions, but the others appear to be spontaneous. Two deletions occurred in intergenic repeat regions, one of which is CRISPR-associated. In the promoter region of gene Tsac_2564 encoding a phosphotransferase subunit, there is a possible transposon insertion. No sequencing reads span the insertion site, but they contain the duplicated sequence ATTTTTAATTATTTT and additional sequence that matches part of the gene Tsac_0046-encoding pyruvate-ferredoxin oxidoreductate (PFOR), a critical gene for ethanol production [19].

**Table 4:** Genomic resequencing results from strains in the present study: large-scale sequence variations and their occurrence in each strain

Locus	Nucleotides	Description	LL1025 (WT)	M863	M1442	M2886
Tsac_0179		Engineered *ldh* deletion	WT	*Deletion*	*Deletion*	*Deletion*
Tsac_0389	424,393–424,493	Small deletion in CRISPR repeat region	WT	*Deletion*	*Deletion*	*Deletion*
Tsac_0832	875,581–875,753	Small deletion in intergenic repeat region	WT	*Deletion*	*Deletion*	*Deletion*
Tsac_1744–1745		Engineered *pta/ack* deletion	WT	*Deletion*	*Deletion*	*Deletion*
Tsac_2564	2618,783–2618,797	Transposon insertion in putative promoter of gene for PTS IIA subunit	WT	*Putative transposon*	*Putative transposon*	*Putative transposon*
Tsac_0795		Engineered deletion	WT	WT	*Deletion*	*Deletion*
Tsac_1474–1477		Engineered deletion of EPS gene cluster	WT	WT	*Deletion*	*Deletion*
Tsac_2114		Engineered *mgs* deletion/insertion	WT	WT	WT	*Breakpoints in 23* *% of reads* ^b^
Tsac_2491		Engineered *perR* deletion	WT	WT	WT	*Deletion*

^a^The *pta/ack* genes were re-introduced elsewhere in the genome

^b^The fraction of the reads supporting the mutation (left and right breakpoints averaged). This value was >90 % for all other breakpoints

For most of the spontaneous mutations in Table 3, it is unknown whether they conferred adaptive phenotypes. Although creation of isogenic strains for each allele is required to rigorously establish genotype:phenotype relationships, inferences about the importance of various mutations may be made based on their recurrence in multiple lineages. Table 5 shows recurrent mutations from all strains resequenced under this project. We observed independent occurrence of mutations in the *adhE* and *hfs* cluster genes as reported previously, along with 11 others. Of particular interest, two sets of mutations occurred in PTS-related transcriptional regulators encoded by Tsac_1263 and Tsac_2568, and another in a PTS IIBC subunit encoded by Tsac_0032. Recurrent mutations in Tsac_0825-encoding inorganic diphosphatase and Tsac_1419-encoding ATPase are also noteworthy for their potential impact on ethanol production. The mutations in Tsac_0361 are also interesting, because the protein encoded by this gene is one of the most abundant secreted proteins and a primary component of the S-layer [20].

**Table 5:** Genomic resequencing results from all strains sequenced in this project: recurrent mutations

Gene	Description	Independent alleles	Present in this lineage
Tsac_0032	PTS system, *N*-acetylglucosamine-specific IIBC subunit	2	Yes
Tsac_0079	Uncharacterised conserved protein UCP018688	2	Yes
Tsac_0361	S-layer domain-containing protein	4	
*adhE* Tsac_0416	Bifunctional alcohol/aldehyde dehydrogenase	4	Yes
Tsac_0644	Hypothetical protein	2	
Tsac_0653	Methionyl-tRNA synthetase	2	
Tsac_0825	Inorganic diphosphatase	2	
Tsac_0838	Protein of unknown function DUF324	3	Yes
Tsac_1263	PTS system transcriptional activator	3	
Tsac_1419	ATPase, F0 complex, subunit A	2	Yes
Tsac_1520	ATP:corrinoid adenosyltransferase BtuR/CobO/CobP	2	
*hfs* Tsac_1550-1553	Hydrogenase large subunit domain protein	8	Yes
Tsac_2568	PTS modulated transcriptional regulator, MtlR family	2	

### Fermentations

**Fig. 1:**
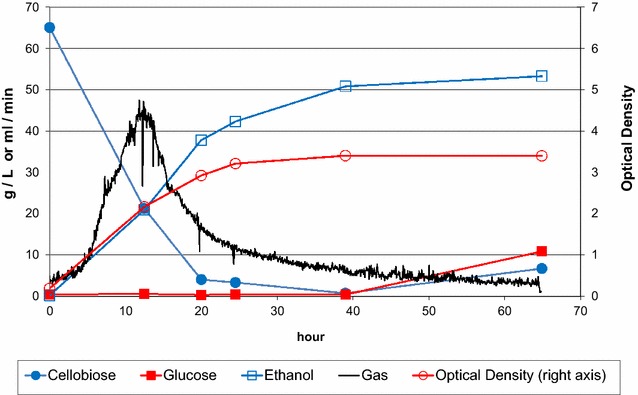
Fermentation of cellobiose and maltodextrin. Strain M1151 was grown in TSC3 medium containing 90 g/L maltodextrin and 60 g/L cellobiose at 1 L scale. Cellobiose, glucose, and ethanol levels are shown in units of g/L on the *left axis*

Fermentation conditions were developed to reach the highest possible ethanol titer with *T. saccharolyticum* in batch format, at 20 mL liquid volume in anaerobic 125 mL serum bottles. These conditions were used to benchmark different strains for ethanol production. We found that cellobiose and starch were readily fermented and well-tolerated at relatively high concentrations. A mixture of 60 g/L cellobiose and 90 g/L maltodextrin in TSC3 rich medium yielded a maximum of 70 g/L ethanol (Table 2). An excess of calcium carbonate provided excellent buffering at a pH of 5.5, which is close to the pH optimum for *T. saccharolyticum*. For reasons we do not fully understand, the same growth media in 1 L fermenters yielded 5–10 g/L less ethanol (Fig. 1).

Fermentation conditions were then developed to reach the highest possible ethanol titer in a Simultaneous Saccharification and Co-Fermentation (SSCF) configuration with substrates approximating the conditions we expected from pre-treated hardwood (i.e., a “mock hydrolysate”). The fermentation contained 100 g/L purified cellulose (Sigmacell-20) and 10 g/L acetic acid, and was fed with 35 g/L xylose and 20 g/L of glucose. We had found that commercially available cellulases were inactivated by low redox and ethanol [11], so we added a mixture of fungal and bacterial cellulase from *C.thermocellum* (see “Methods” section). The *T. saccharolyticum* inoculum was drawn from a chemostat, so that it was active and had a consistently high optical density (5–10 OD units). The results of this fermentation are shown in Table 6, comparing the previously published strain ALK2 to the improved strain M1442. An ethanol titer of 61 g/L was reached in 93 h by strain M1442 while strain ALK2 produced 46 g/L, leaving some residual xylose. The metabolic yield for both strains was greater than 90 % of the theoretical maximum, while the cellulose conversion by the enzyme mix was 71–75 %. Scaled up to 8 L, strain M1442 produced 55 g/L ethanol.

**Table 6:** Fermentation data comparing strains ALK2, M1442, and M2886 in pH-controlled bioreactors in SSCF or SHF process configurations

Substrate	Mock hydrolysate	Mock hydrolysate	Mock hydrolysate	Pre-treated hardwood	Pre-treated hardwood	Pre-treated hardwood	Hardwood hydrolysate
Process type	SSCF	SSCF	SSCF	SSCF	SSCF	SSCF	SHF
Fermentation volume (liters)	1	1	8	1	1	100	1
Strain	ALK2	M1442	M1442	ALK2	M2886	M2886	M2886
Initial concentrations							
Solids (%)	16.5	16.5	16.5	12.0	12.0	12.0	0.0
Cellulose (g/L)	100	100	100	64.5	64.5	64.5	0.0
Glucose (g/L)	20	20	20	1.0	1.0	1.2	88.6
Xylose (g/L)	35	35	35	13.9	13.9	16.6	24.3
Other sugars (g/L)				3.8	3.8	3.2	5.2
Acetic acid and other inhibitors (g/L)	10.5	10.5	10.0	6.3	6.3	6.3	4.5
Fermentation performance							
Fermentation time (hours)	97	93	90	60	60	60	60
Final ethanol titer (g/L)	45.7	61.4	54.7	32.6	32.0	30.8	49.5
Cellulose conversion (%)	71.0	75.1	83.4	83.6	80.4	77.2	n/a
Glucose utilization (%)	79.5	93.6	85.9	97.2	94.7	99.5	89.3
Xylose utilization (%)	58.1	99.6	80.9	82.3	86.7	100.0	91.6
Metabolic yield (%)	90.5	91.5	85.8	75.9	81.0	78.4	90.1

**Fig. 2:**
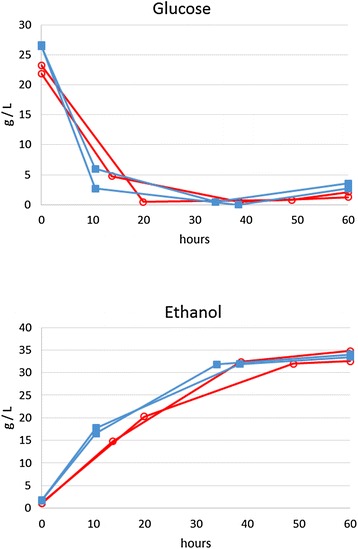
Fermentation of 12 % solids pre-treated hardwood, fed with hemicellulose extract. Duplicate 1 L fermentations with strain ALK2 are shown in *red* with *open circles* and with strain M2886 in *blue filled squares*

An SSCF was also performed with pre-treated hardwood at 12 % solids concentration, comparing two strains in duplicate. A concentrated, polymeric hemicellulose extract was fed, and activated carbon was used to reduce the toxicity of both the solids and the liquid feed. Again, a mixture of fungal and *C. thermocellum* cellulases was used, and cellulose conversion was 80–84 %. Strain M2886 produced 32 g/L ethanol in 60 h, while ALK2 produced 33 g/L ethanol, at 81 and 76 % of theoretical metabolic yield, respectively. Scaled up to 100 L, strain M2886 produced 31 g/L ethanol. Other fermentations at 22 % solids loading performed poorly (not shown), likely due to the presence of inhibitors at levels higher than the cells could tolerate. At 12 % solids, there was a little difference in performance between the project’s starting and final strains (ALK2 and M2886, respectively, Fig. 2), while at 22 % solids, both strains were inhibited. We can speculate that at some intermediate level of solids loading, inhibition would be enough to better distinguish the performance of the two strains, but not too much for M2886 to grow. Figure 2 shows that at approximately 40 h, the glucose levels in all fermentations were below 1 g/L and ethanol was greater than 30 g/L, suggesting that the cultures were limited by the availability of glucose (i.e. the activity of the cellulases) at that time. Some glucose accumulated by 60 h, suggesting that cellulase-mediated solubilization rates exceeded the rate of fermentation.

To demonstrate the ability of *T. saccharolyticum* to produce high ethanol titers when cellulase activity is not limiting, a separate hydrolysis and fermentation (SHF) was performed with pre-treated hardwood hydrolysate and hemicellulose extract (last column of Table 6). After 60 h of fermentation, the ethanol titer reached 50 g/L, while sugar utilization and metabolic yield were 90 %.

*Thermoanaerobacterium saccharolyticum* is distinct from other homoethanologens in its native ability to digest polymeric hemicellulose and to co-ferment all the resulting sugars at high ethanol yield. Commercial bioprocessing configurations can be considered where hemicellulose is separated from biomass by hot water extraction and fermented separately. *T. saccharolyticum* would be a good choice of organism for such fermentations, because it can mediate hydrolysis of the polymeric hemicellulose without added enzymes or acid, though it needs to be able to handle the acetic acid and other inhibitors that normally accompany it. Some level of detoxification can be considered, but the cost must be kept very low.

**Fig. 3:**
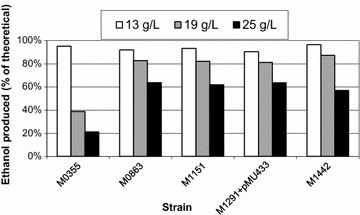
Strain comparison in varying levels of hemicellulose extract. *Bars* indicate ethanol produced in batch fermentations as a percent of the maximum theoretical ethanol possible for the amount of sugar provided by the extract, indicated by *bars*: 13 g/L (*white*), 19 g/L (*gray*), and 25 g/L (*black*)

**Fig. 4:**
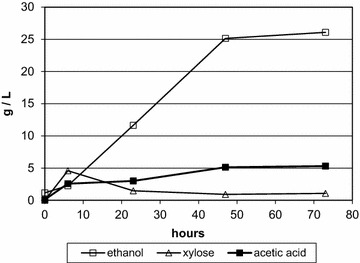
Fermentation of detoxified hemicellulose extract. Strain M1732 was grown in TSC7 medium containing hemicellulose extract at 1 L volume at 51 °C at pH 5.8. The fermentor contained 42 g/L available sugar (76 % xylose, 11 % mannose, 6 % glucose, 5 % galactose, and 2 % arabinose, as polymeric hemicellulose) at the start, and was fed an additional 25 g/L over two feedings at 24 and 47 h. The hemicellulose was detoxified by lime treatment and nanodiafiltration

A number of strains were evaluated at varying levels of hemicellulose extract, as shown in Fig. 3. At low concentrations of extract (13 g/L total sugar), the ethanol yields exceeded 90 %, but the yields declined rapidly at higher concentrations of extract. Lime treatment and nanofiltration were used to detoxify the extract, which was fermented in fed-batch at 1 L scale (Fig. 4). After 47 h, 25 g/L of ethanol was produced, and increased to 26 g/L by 73 h. Xylose, the main sugar component, was low throughout the fermentation, and arabinose was undetectable by 23 h. The final metabolic ethanol yield was 78 % of theoretical.

It has been noted in the literature that tolerance to added ethanol is often higher than the maximum titers of ethanol that are produced, but this ‘gap’ can be eliminated by strain adaption and engineering [21]. The maximum titer of produced ethanol reported here (70 g/L) is consistent with reports for the maximum concentrations of added ethanol at which thermophilic anaerobes will grow after selection for ethanol tolerance—generally in the range of 50–70 g/L [22]. Thus, the strain and pathway reported here represent a new example of success in closing the titer gap among thermophilic ethanol producers. Production of ethanol beyond the maximum at which growth occurs is possible based on uncoupled metabolism, although this has received relatively little study in thermophiles to date. The ethanol tolerance of thermophilic strains selected for growth in the presence of ethanol is similar to that described for engineered strains of *E. coli*, but not as high as either the bacterium *Zymomonas mobilis* or *Saccharomyces cerevisiae*. Higher ethanol titers can be achieved for a given species or strain at lower temperatures within its growth range [23], but we have no reason to think that an interspecies comparison between thermophiles and mesophiles would show the same trend. It should be noted, however, that beyond approximately 40 g/L, ethanol titer has a diminishing effect on distillation costs, and lignocellulosic materials are difficult to convert to ethanol at much more than 50 g/L due to inherent limitations such as mixability and the fraction of fermentable sugar [1, 2].

## Conclusions

Production of ethanol at greater than 90 % yield and at titers greater than 60 g/L from model cellulosic substrates were demonstrated using *T. saccharolyticum* in an SSCF configuration in the presence of 10 g/L acetate. However, maximum ethanol titers were lower using steam pre-treated hardwood or hemicellulose extract. The complex inhibitors present in pre-treated wood are problematic for *T. saccharolyticum* above moderate concentrations. Random and directed strain modifications, along with detoxification steps, have made improvements in increasing substrate tolerance, but not enough to fully overcome the problem. Further work will be needed to analyze what compounds or combinations of compounds are actually inhibitory, or to more fully detoxify the material in a cost-effective way. Alternately, these inhibitors could be simply avoided by elimination of pre-treatment from the bioprocess. The provision of sufficient cellulase activity for *T. saccharolyticum* to be used in SSF has proved to be problematic with existing technology. Development of a bacterial lignocellulose solubilization system and/or an understanding of the limitations of fungal cellulases at low-redox levels are necessary for the further development of *T. saccharolyticum* as biocatalyst for SSF of pre-treated hardwood. However, the high titers and yields we observed support the feasibility of using engineered thermophiles for industrial ethanol production if challenges associated with pre-treatment inhibitors can be avoided.

## Methods

### Plasmids, primers, and genetic engineering

All markerless gene knockouts were performed as described earlier [13]. The chromosomal flanking regions were PCR amplified with primers listed in Table 7. These PCR products were fused to plasmid pMU433 to create the following gene knockout plasmids: pMU1546 targeting the EPS cluster, including gene Tsac_1474-Tsac_1477; pMU1301 targeting the *perR* gene Tsac_2491; and pMU3014 targeting the *mgs* gene Tsac_2114.

**Table 7:** Oligonucleotide primers

Primer	Description	Sequence
X04986	perR up-stream forward primer	tttcgactgagcctttcgttttatttgatgcctggTTTGTAATAAAGTCTGCCGT
X04987	perR up-stream reverse primer	AATTGTAGAATACAATCCACTTCACAATGGGCACGTTTTCTTTCAGGATTGACGA
X04989	perR down-stream reverse primer	CCGTCAGTAGCTGAACAGGAGGGACAGCTGATAGAGGCGATAAAGACTATGTAGA
X05122	perR down-stream forward primer	aggggtcccgagcgcctacgaggaatttgtatcgCACAGATTACCTTTTGATGG
X07562	EPS up-stream forward primer	tttcgactgagcctttcgttttatttgatgcctggccgaaaggataagagagcttgc
X07563	EPS up-stream reverse primer	AATTGTAGAATACAATCCACTTCACAATGGGCACGGCATGATGAGGCGATACCTTGATG
X07564	EPS down-stream forward primer	aggggtcccgagcgcctacgaggaatttgtatcggttcctgataaacctgtatcgccc
X07565	EPS down-stream reverse primer	CCGTCAGTAGCTGAACAGGAGGGACAGCTGATAGACTGCCAGCGATGTAAAGCATAG
X07568	EPS external primer 1	acttggatacaggcagtggaggaa
X07569	EPS external primer 2	TCCAGCATAGCCTGCAACTGGATA
X13281	perR external primer 1	agctatgctttctacccttgccca
X13282	perR external primer 2	AACGACAAGCAGTTTGTGCTTCCG
X15225	mgs up-stream forward primer	agcttgatatcgaattcctgcagcccgggggatctCAGTGCGTCACACGCAGTTG
X15226	mgs up-stream reverse primer	agaatacaatccacttcacaatgggcacgGGATCCGATCTTTTGCCTTCGCATCCC
X15227	mgs down-stream forward primer	gtcccgagcgcctacgaggaatttgtatcgGATCCGGATTTTTGGAATGGAGAGATG
X15228	mgs down-stream reverse primer	accgcggtggcggccgctctagaactagtGGATCTGGTCCTGCTAATGCGATGATG
X15767	mgs external primer 1	TGCACATTCAGTGCCGTTGTC
X15768	mgs external primer 2	GTAATCCAACTGAGTGCCGATG

### Classical mutagenesis and selection

An enzymatic hydrolysate was prepared to serve as substrate for mutagenized cultures. Pre-treated hardwood was hydrolyzed with 30 mg/g Accellerase (DuPont) cellulase in a 10 L bioreactor at 10 % initial solids and subsequently fed additional solids up to 20 %. The bioreactor temperature was 50 °C and the pH was 4.8. After 5 days of hydrolysis, the enzymes were heat inactivated at 80 C for 1 h, and the liquids were filtered with Whatman Shark Skin filter paper to remove solids, and then filter sterilized. *T. saccharolyticum* was mutagenized with 100–160 ppm nitrosoguanidine for 30–60 min at Panlabs Biologics (Taiwan), then diluted and cultured on petri plates in an anaerobic chamber to isolate clones. The clones were screened by culturing in tubes containing BA medium, 1–19 g/L each of xylose, glucose, and/or cellobiose, and up to 25 % volume of enzymatic hydrolysate. HPLC was used to measure ethanol production and substrate utilization, and the best clones were chosen for additional rounds of mutagenesis and screening.

### Library construction

A Gateway Cloning (Life Technologies, Carlsbad, CA) destination vector called pMU1035 was constructed with the cellobiose phosphorylase promoter from *C. thermocellum* positioned up-stream from a cloning site and a *ccdB* cassette for negative selection. Adjacent to these were sequences flanking the *T. saccharolyticumldh* gene, chosen as the site for chromosomal integration. It was constructed by inserting the cellobiose phosphorylase promoter between the up-stream *ldh* flanking region and the kanamycin resistance gene in plasmid pMU433 [13] using yeast-mediated ligation [24]. The resulting plasmid was digested with the enzyme *Sna*BI and a PCR product containing the *ccdB* gene was ligated. A library of randomly cleaved genomic DNA from *T. saccharolyticum* was cloned first into the pCR8/GW/Topo entry plasmid and then transferred into pMU1035 by a clonase LR reaction. The reaction mix was transformed into *E. coli* strain Mach1 (Life Technologies) and selected for kanamycin resistance, generating the overexpression library. Plasmid DNA from this library was used to transform *T. saccharolyticum* and selected for kanamycin resistance before being used in growth selection experiments.

The *T. saccharolyticum* knockout library was generated by modifying the previously created overexpression library. Briefly, the overexpression library was digested with a set of three restriction enzymes that frequently cut *T. saccharolyticum* genomic DNA but do not cut anywhere on the cloning vector backbone. The kanamycin resistance gene was ligated into the digested library, transformed into *E. coli*, and 2000–6000 kanamycin-resistant colonies were collected for each of the enzymes used. This produced a large number of plasmids containing the kanamycin resistance marker flanked by *T. saccharolyticum* genomic DNA on either side, which were transformed and integrated into the *T. saccharolyticum* genome. These transformants were selected for kanamycin resistance, then screened or selected for inhibitor tolerance. To identify the overexpressed or knockout gene, genomic DNA was isolated and cloned into an *E. coli* plasmid vector and selected for kanamycin resistance. The resulting colonies were then sequenced.

### Resequencing

Raw data for strain M863 were generated at the National Center for Genome Resources (Santa Fe, NM) using an Illumina Solexa Genome Analyzer. The data comprised single 36 bp reads (non-paired).

Raw data for strains M1442 and wild-type JW/SL-YS485 were generated by the Joint Genome Institute (JGI) with an Illumina MiSeq instrument as described by Zhou and coworkers [19]. Unamplified libraries were generated using a modified version of Illumina’s standard protocol. 100 ng of DNA was sheared to 500 bp using a focused ultrasonicator (Covaris). The sheared DNA fragments were size selected using SPRI beads (Beckman Coulter). The selected fragments were then end repaired, A tailed, and ligated to Illumina compatible adapters (IDT Inc.) using KAPA- Illumina library creation kit (KAPA biosystems). Libraries were quantified using KAPA Biosystem’s next-generation sequencing library qPCR kit and run on a Roche LightCycler 480 real-time PCR instrument. The quantified libraries were then multiplexed into pools for sequencing. The pools were loaded and sequenced on the Illumina MiSeq sequencing platform utilizing a MiSeq Reagent Kit v2 (300 cycle), following a 2 × 150 indexed run recipe. Paired-end reads were generated, with an average read length of 150 bp and paired distance of 500 bp.

Raw data for strain M2886 were generated at the Oak Ridge National Laboratory. Illumina TruSeq libraries were prepared as described in the manufacturer’s methods (Part# 15005180 RevA) following the low throughput protocol. In short, 3 ug of DNA was sheared to a size between 200 bp and 1000 bp by nebulization using nitrogen gas for 1 min at 30 psi. Sheared DNA was purified on a Qiagen Qiaquick Spin column (Qiagen). The sheared material was assessed for quantity with a Qubit broad range double stranded DNA assay (Life Technologies) and quality by visualization on an Agilent Bioanalyzer DNA 7500 chip (Agilent). One microgram of sheared DNA was used for library preparation following the manufacturer’s protocol. Libraries were validated by Qubit (Life Technologies) and Agilent Bioanalyzer for appearance and size determination. Samples were normalized using Illumina’s Library dilution calculator to a 10 nM stock and diluted further for sequencing. Clustering was completed on an Illumina CBot, and paired-end sequencing was completed on an Illumina HiSeq instrument (101 bp for each end and 7 bp for the index) using TruSeq sequencing-by-synthesis chemistry.

Data analysis was performed using CLC Genomics Workbench, version 8.5 (Qiagen, USA). Reads were mapped to the reference genome (NC_017992). Mapping was improved by two rounds of local realignment. The CLC probabilistic variant detection algorithm was used to determine small mutations (single and multiple nucleotide polymorphisms, short insertions, and short deletions). Variants occurring in less than 90 % of the reads and variants that were identical to those of the wild-type strain (i.e., due to errors in the reference sequence) were filtered out. The fraction of the reads containing the mutation is shown in Table 3. To determine larger mutations, the CLC InDel and Structural Variant algorithm was run. This tool analyzes unaligned ends of reads and annotates regions where a structural variation may have occurred, which are called breakpoints. Since the read length averaged 150 bp and the minimum mapping fraction was 0.5, a breakpoint can have up to 75 bp of sequence data. The resulting breakpoints were filtered to eliminate those with fewer than ten reads or less than 20 % “not perfectly matched.” The breakpoint sequence was searched with the Basic Local Alignment Search Tool (BLAST) algorithm for similarity to known sequences [25]. Pairs of matching left and right breakpoints were considered evidence for structural variations, such as transposon insertions and gene deletions.

### Media and bottle cultures

Growth media were prepared as 10× concentrates and filter sterilized, then immediately added to fermenters or stored in sterile, nitrogen-flushed serum bottles. Chemicals were from Sigma-Aldrich (St. Louis, MO, USA). The medium TSC3 at 1× concentration contained: 8.5 g/L yeast extract, 4 g/L trisodium citrate dihydrate, 2.0 g/L monobasic potassium phosphate, 2.0 g/L magnesium sulfate heptahydrate, 5 g/L urea, 0.2 g/L calcium chloride dihydrate, 0.1 g/L iron sulfate heptahydrate, 0.12 g/L l-methionine, and 0.5 l-cysteine hydrochloride. Medium TSC6 at 1× concentration contained: 8.5 g/L yeast extract, 0.5 g/L trisodium citrate dihydrate, 2.0 g/L monobasic potassium phosphate, 2.0 g/L magnesium sulfate heptahydrate, 5 g/L urea, 0.2 g/L calcium chloride dihydrate, 0.2 g/L iron sulfate heptahydrate, 0.12 g/L l-methionine, and 0.5 l-cysteine hydrochloride. Medium TSC7 at 1× concentration contained: 8.5 g/L yeast extract, 1.0 g/L trisodium citrate dihydrate, 1.0 g/L monobasic potassium phosphate, 2.0 g/L magnesium sulfate heptahydrate, 1.85 g/L ammonium sulfate, 0.2 g/L calcium chloride dihydrate, 0.2 g/L iron sulfate heptahydrate, 0.12 g/L l-methionine, and 0.5 l-cysteine hydrochloride. The medium BA at 1× concentration contained: 3 g/L trisodium citrate dihydrate, 1.5 g/L monobasic potassium phosphate, 2.4 g/L magnesium sulfate heptahydrate, 2 g/L ammonium sulfate, 0.2 g/L calcium chloride dihydrate, 0.1 g/L iron sulfate heptahydrate, 0.015 g/L l-methionine, 0.02 g/L para-amino benzoic acid, 0.02 g/L thiamine, and 0.0001 g/L vitamin B12.

Bottle cultures were performed in 125 ml serum bottles sealed with blue butyl rubber stoppers and crimp seals. Culture volumes were 20 or 50 ml in 125 ml bottles, and those with high sugar concentrations were vented periodically to prevent hazardous pressure build-up. Sugars were dissolved in de-ionized water, and calcium carbonate was added to a final concentration of 10 g/L. The bottles were sealed and then flushed with a 5 % carbon dioxide, 95 % nitrogen gas mixture. They were incubated at 51–55 °C in an incubator shaking at 125–150 rpm. In Fig. 3, cultures were performed in anaerobic tubes with 5 ml liquid volume, using TSC6 medium with 15 g/L calcium carbonate and 1.85 g/L ammonium sulfate in place of urea as nitrogen source. The hemicellulose extract was concentrated by evaporation and analyzed by quantitative saccharification analysis. Inoculations for Fig. 3 were 10 % of the total volume.

### Cytostat

To adapt *T. saccharolyticum* to rapid growth in a mixture of inhibitors found in pre-treated hardwood, a cytostat was constructed and operated as per [14]. The medium used for continuous cultivation of *T. saccharolyticum* contained (per liter): 20 g ethanol, 24 mg gallic acid, 395 mg hydroxymethylfurfural, 405 mg furfural, 95 mg 3,4-dihydroxybenzoic acid, 19 mg syringic acid, 37 mg vanillin, and 61 mg syringaldehyde.

### Fermentations

Fermentations were conducted in 2 L Biostat A reactors (Sartorius AG, Goettingen, Germany) at 1 L working volume. Sugars or pre-treated hardwood along with 10 g/L calcium carbonate and 10 g/L Norit PAC200 activated carbon were added to de-ionized water, and the fermenters were autoclaved. They were sparged with a 5 % carbon dioxide, 95 % nitrogen gas mixture while cooling to fermentation temperature of 51–55 °C. Medium TSC7, prepared at 10× concentration, was filter sterilized and added to the reactors. The pH was set to 5.5 with ammonium hydroxide. Before inoculation of SSFs, cellulase was added for 3–5 h of prehydrolysis. An inoculum of 100 ml was added from a chemostat maintained at a dilution rate of 0.1 h^−1^ with TSC7 medium with 38 g/L glucose plus 11 g/L total sugars in extract from pre-treated hardwood, at pH 5.8 and 55 °C. For the SSCF fermentations shown in Fig. 2, a feed of 80 mL of activated carbon-treated and dialyzed hemicellulose extract was started after inoculation and 90 mL of *C. thermocellum* cellulase was added.

SHF fermentations were performed as fed-batch in duplicate, feeding a mixture of liquid solutions prepared from pre-treated hardwood. Polymeric hemicellulose (mostly 5-carbon sugars) was extracted from pre-treated hardwood, treated with lime and activated carbon, and concentrated with nanofiltration. The water-washed solid pre-treated hardwood (mostly 6-carbon sugars) was enzymatically digested with fungal cellulase, concentrated, and treated with activated carbon. The two preparations were mixed in proportion to the abundance of sugars in unfractionated pre-treated hardwood. Glucose levels in the fermentation were monitored carefully and feed rate adjusted to keep the glucose levels less than 0.5 g/L, which we had determined to be important for optimizing ethanol production.

### Cellulases

The SSCF of Sigmacell-20 (a purified cellulose sold by Sigma-Aldrich, St. Louis, MO) shown in Table 6 was conducted with 10 mg enzyme per gram of dry solids using a 3:1 mixture of monocomponent CBHI and Endoglucanase from AB Enzymes (Darmstadt, Germany). The SSCF of pre-treated hardwood shown in Fig. 2 was conducted with 20 mg/g CTec3 from Novozymes (Bagsvaerd, Denmark). To supplement fungal cellulases, bacterial cellulase was prepared by growing *C. thermocellum* strain ATCC 27405 on 5 g/L avicel until early stationary phase. The culture broth was left to settle overnight at 4 °C, and then decanted. The supernatant was concentrated 5- to 10-fold using a 500 kDa filter in tangential flow filtration, then frozen until needed. Before use, cellulosome preparations were centrifuged briefly then filter sterilized. Fungal cellulases were stored at 4 °C and bacterial cellulase was stored at −20 °C.

### HPLC

Fermentation products and residual sugars were acidified with sulfuric acid and analyzed using an Aminex HPX-87H (300 × 7.8 mm) column (Bio-Rad Laboratories, Hercules, CA, USA), protected by an in-line frit (0.2um) and Cation-H guard column. Analytes were detected by refractive index and optional UV detector. Eluent was 5 mM sulfuric acid diluted in de-ionized water and the flow rate was 0.7 mL/min at 65 °C.

## Abbreviations

SSF: simultaneous saccharification and fermentation
SSCF: simultaneous saccharification and co-fermentation
SHF: separate hydrolysis and fermentation
DNA: deoxyribonucleic acid
PCR: polymerase chain reaction
EPS: exopolysaccharide
HPLC: high-performance liquid chromotography
UV: ultraviolet
